# A reusable PMMA/paper hybrid plug-and-play microfluidic device for an ultrasensitive immunoassay with a wide dynamic range

**DOI:** 10.1038/s41378-020-0143-5

**Published:** 2020-06-15

**Authors:** Sharma T. Sanjay, Meihan Li, Wan Zhou, Xiaochun Li, XiuJun Li

**Affiliations:** 1 Department of Chemistry and Biochemistry, University of Texas at El Paso, 500 West University Ave, El Paso, TX 79968 USA; 2 College of Biomedical Engineering, Taiyuan University of Technology, 030024 Taiyuan, Shanxi China; 3 Border Biomedical Research Center, Biomedical Engineering, and Environmental Science and Engineering, University of Texas at El Paso, 500 West University Ave, El Paso, TX 79968 USA

**Keywords:** Chemistry, Engineering

## Abstract

Conventional colorimetric enzyme-linked immunosorbent assay (ELISA) is a time-consuming laboratory assay that is not very sensitive and consumes a large amount of samples. Herein, the development of a reusable, cost-effective, and eco-friendly poly(methyl methacrylate) (PMMA)/paper hybrid plug-and-play (PnP) device for high-sensitivity immunoassay by analyte enrichment and efficient passing-through washing has been reported. The PMMA device has multiple slots where a pre-patterned paper substrate can be inserted. The sample flows back-and-forth through a low-cost, 3D paper substrate within the PMMA channels, thereby enhancing the amount of analyte adsorbed and dramatically increasing the sensitivity while decreasing the assay time. After the enrichment assay, the paper substrate can simply be pulled out of the device, and the results can be qualitatively viewed with the naked eye or scanned through a simple desktop scanner for quantitative analysis. The paper substrate can be replaced with a new substrate so that the device can be reused. The limits of detection (LODs) of 200 pg/mL for immunoglobulin G (IgG) and 270 pg/mL for hepatitis B surface antigen (HBsAg) were obtained. This IgG assay is at least 10 times more sensitive than commercial ELISA kits. In addition, the PnP ELISA exhibited a significant increase in the linear dynamic range from 3 orders of magnitude in a common paper-based device to a wide range of six orders of magnitude in the PnP hybrid device. This reusable PnP device has great potential for the low-cost yet high-sensitivity detection of infectious diseases, cancers, and other important biomolecules.

## Bioanalysis: Simple, eco-friendly, portable PnP device for ultrasensitive immunoassays

A reusable, cost-effective, and environment-friendly PnP device has been developed for high-sensitivity immunoassays (biochemical tests that measure proteins or other substances through their properties as antigens or antibodies). Immunoassays are widely used in the diagnosis, screening, and monitoring of diseases. However, such techniques as enzyme-linked immunosorbent assay (ELISA), which are extensively applied in developed countries, are not widely available in developing nations owing to limited funding and lack of skilled manpower. They are also time consuming and lacking in sensitivity. A team headed by XiuJun Li at the University of Texas at El Paso produced a low-cost device using poly(methyl methacrylate) (PMMA or Perspex) that is 10 times more sensitive than commercial ELISA kits. The authors believe that their PMMA device offers considerable potential for application in detecting infectious diseases and cancers in resource-poor settings.

## Graphical abstract

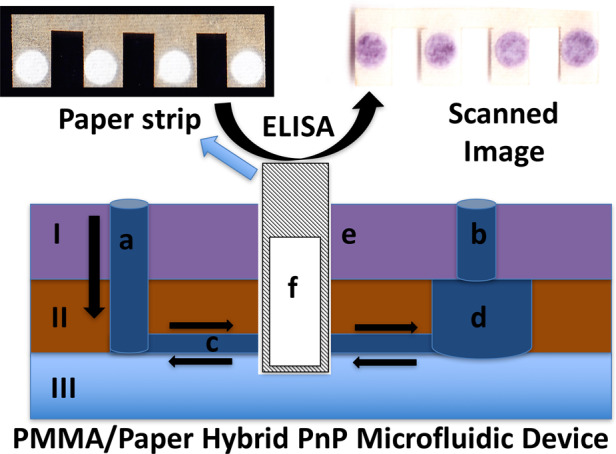

## Introduction

Infectious diseases caused by bacteria, viruses or fungi lead to approximately 13.2 million deaths, which is 25% of the deaths worldwide and accounts for more than half of all infant deaths^1,2^. In addition, 95% of these deaths are due to the lack of cost-effective medical interventions^3^. Although advanced and sophisticated diagnostic technologies, including polymerase chain reaction (PCR) and enzyme-linked immunosorbent assay (ELISA), are extensively used in developed countries, they are not widely available in developing countries because of limited funds and a lack of skilled technicians. These techniques, along with other methods, such as cell culture, western blotting, and flow cytometry, are often laborious and time-consuming. Hence, point-of-care (POC) detection platforms are in great need in resource-limited settings^4–7^.

Although ELISAs performed in 96-well plates are one of the most commonly used laboratory methods, they require overnight incubation, consume a large volume of valuable samples and expensive reagents, and require specialized personnel and laboratory settings with bulky and expensive robotic pipettors, plate washers, and optical detectors^8,9^. Likewise, the other major problem of colorimetric ELISA is the low detection sensitivity for precious, low-volume and low-concentration analytes, especially for real-world samples. This technique may not be sensitive enough to detect some low-concentration analytes that typically have cutoff values below 1 ng/mL^10^. For instance, low serum levels of procalcitonin (<0.35 ng/mL) are associated with decreased severity of lower respiratory tract infections, and patients can be safely managed outside intensive care units (ICU), while patients having procalcitonin serum levels >0.35 ng/mL need to be admitted to the ICU^11,12^. New, low-cost yet ultrasensitive ELISA methods need to be developed to address those problems^13^.

Microfluidic devices fabricated by microelectromechanical system (MEMS) technology incorporate different fields, including electronics, mechanics, optics, biological detection, and sensors, to address problems in biomedical applications^14–16^. Microfluidic lab-on-a-chip (LOC) devices reduce the amount of samples and reagents required, integrate multiple functionalities on a single device, and provide high portability^17–19^. Paper-based microfluidic devices that do not require cleanroom fabrication further reduce the cost of disease diagnosis and have been widely used for POC analysis^20–23^. Colorimetric paper-based devices are the most widely employed detection platforms since the paper substrate offers a bright, high-contrast, and colourless background for colour change readings^21,24,25^. For instance, Whitesides’ group performed colorimetric ELISA in a 96-microzone paper-based device^21^. Although the assay could be completed within an hour, it was not as sensitive as the conventional ELISA, where the lowest IgG concentration detected was 670 nM (i.e., 100 µg/mL). Murdock et al. performed paper-based enzyme-free ELISA for the assay of Neuropeptide Y and IgG, but the assay required complicated and time-consuming conjugation with gold nanoparticles, and the LOD for IgG was ~10 nM (i.e., 1500 ng/mL)^24^. Our group developed a paper-based microfluidic microplate for disease biomarker detection using a common office scanner and achieved an LOD of 1.6 ng/mL for IgG. Because of the advantage of the high surface-to-volume ratio of porous paper^26^, antibodies were rapidly immobilized in those assays (usually within minutes)^20^, compared to the hours or overnight incubation required for conventional ELISA. However, the LODs of most paper-based colorimetric ELISAs range from 2 ng/mL to 2 µg/mL (for IgG herein). New strategies are needed to improve the sensitivity of these assays to detect low-concentration biomolecules.

Poly(methyl methacrylate) (PMMA) is among the most commonly used polymer substrates in LOC devices due to its low cost of manufacturing and ease of use and fabrication. Likewise, it is transparent, rigid, and can rapidly transfer reagents. PMMA is not advantageous in colorimetric detection but in other optical detection methods, such as fluorescence. However, PMMA requires complicated surface modification for the immobilization of proteins and immune sensors^27–29^. For example, Darain et al. activated the PMMA surface with O_2_ plasma and functionalized it with APTES for stable antibody immobilization^27^. The on-chip ELISA was detected using fluorescence microscopy with an LOD of 0.12 µg/mL. Yu et al. developed a QD-linked immune-diagnostic assay in which myeloperoxidase antibodies were covalently linked to PMMA after treatment with polyethylene glycol followed by glutaraldehyde and required an expensive fluorescence setup for detection^28^. All of these assays require complicated and time-consuming functionalization along with bulky and expensive detection systems and cannot be used in POC settings.

Each microfluidic substrate has its own nobilities and drawbacks. The combination of different substrates can enable the integration of different functionalities, taking advantage of different materials and excluding some limitations of certain materials. Recently, hybrid microfluidic devices have been broadly applied in diverse applications, including the detection of infectious diseases and cellular studies^20,30–32^. Dou et al. developed a PDMS/paper hybrid device for the detection of infectious diseases^33^. They found that the hybrid device provided stable results for a longer period of time compared to a paper-free microfluidic system. Our group also developed a paper/polymer hybrid microfluidic microplate for the detection of multiple disease biomarkers^25^. The use of porous paper in the flow-through microwells of the hybrid system assisted in the rapid immobilization of proteins and efficient washing, mitigating the requirement of sophisticated surface modifications. The device only had an LOD of 1.6 ng/mL IgG and could not be used for the detection of analytes with lower concentrations.

In response to the aforementioned challenges, we have developed a simple and reusable PMMA/paper hybrid plug-and-play (PnP) microfluidic device for ultrasensitive colorimetric immunoassay with a wide dynamic range. The PMMA substrate has multiple laser-ablated slots, where a pre-patterned 3D microporous paper strip can be plugged in. The sample flows back-and-forth through the paper within the PMMA channel, hence increasing the amount of analyte immobilized and decreasing the incubation time. After analyte enrichment on the 3D matrix of the paper, the paper substrate can simply be pulled out of the device to view the results, and a new paper substrate can be plugged in for the next assay. ELISA of immunoglobulin G (IgG) and hepatitis B surface antigen (HBsAg) was performed with the PnP hybrid microfluidic device, and limits of detection (LODs) of 200 pg/mL and 270 pg/mL were obtained, respectively, without the use of any specialized detection system, such as a spectrophotometer/microplate reader. Compared to regular paper-based and PMMA microfluidic devices, our hybrid method exhibited significantly improved sensitivity and a wide linear range of five orders of magnitude for IgG and six orders of magnitude for HBsAg. The sensitivity of the PnP device was at least 10-fold better than that of commercial ELISA kits^10^.

## Results and discussions

### Hybrid PnP microfluidic device

**Fig. 1:**
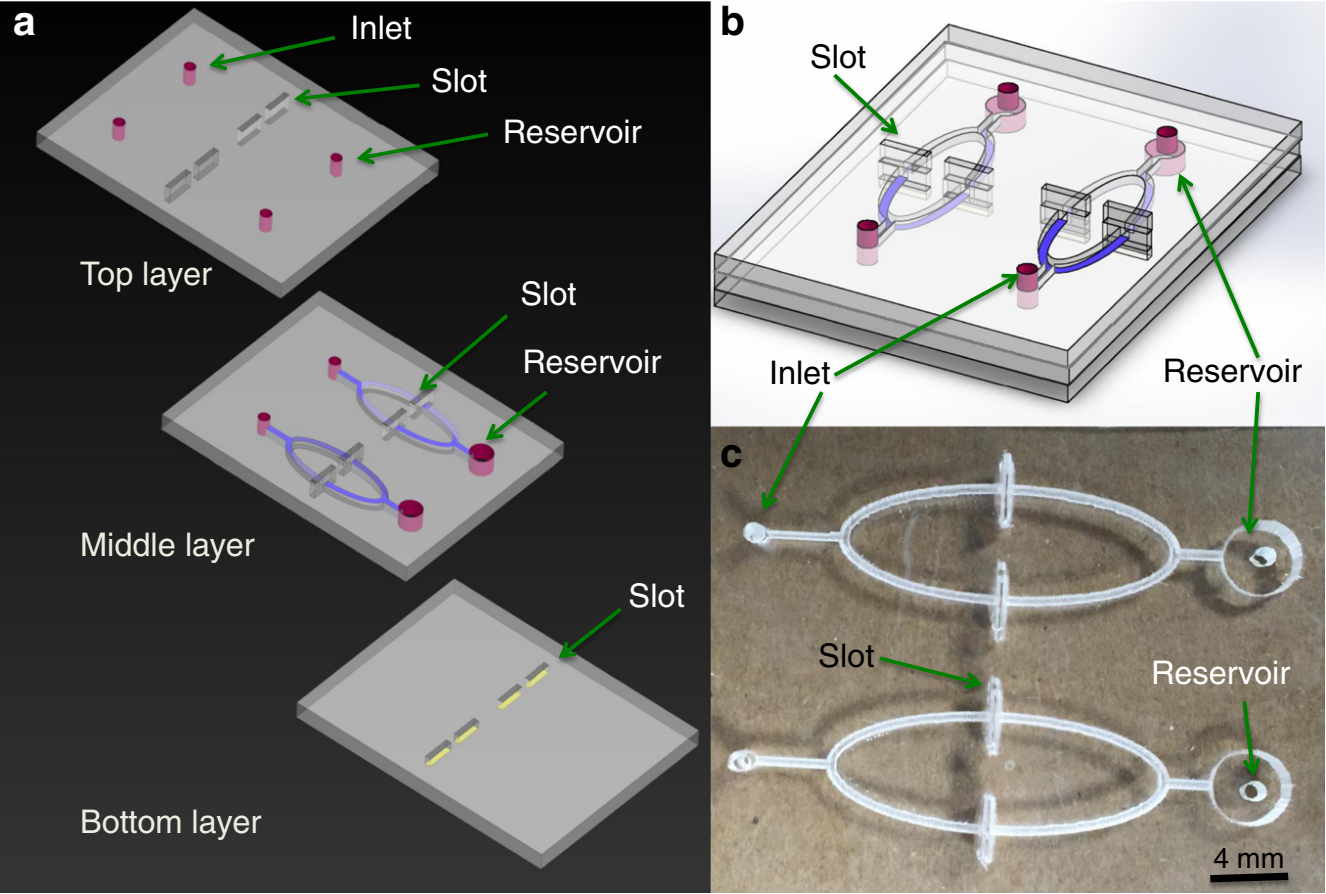
Chip design of the paper/PMMA hybrid PnP device. **a** 3D schematic of the exploded PnP hybrid microfluidic device with the top, middle, and bottom layers shown. **b** 3D view of the assembled PnP hybrid microfluidic device. **c** Photograph of an assembled PnP hybrid microfluidic device.

The PnP hybrid microfluidic device was designed to meet three specific purposes: (i) generation of multiple slots with interconnected channels and a temporary reservoir, where the flow of the analyte through the paper strips could form a loop/cycle for maximum enrichment on the paper substrate; (ii) the ability to reduce background noise and increase sensitivity through efficient washing of the paper substrate; and (iii) utilization of a paper substrate that can be plugged into and pulled out of the device to perform the assay and replaced with a new one for another assay (i.e., PnP). Figure 1 shows the layout of the paper/PMMA PnP microfluidic device designed under the above guidelines. The PnP hybrid device consists of three layers, as shown in the 3D exploded schematic of the device in Fig. 1a. The top and middle layers consist of two inlet microwells, four slots, and two micro-reservoirs. In the middle layer, inlet microwells are connected to the reservoir microwells by two sets of microchannels that pass through two slots. Two slots from one inlet allow for duplicate assays, while two separate units from two different inlets enable multiplexed detection or two additional replicates of the PnP assay. The bottom layer consists of four half-cut slots. Figure 1b shows a 3D view of the assembled PnP hybrid microfluidic device, while Fig. 1c shows a photograph of the actual assembled PnP hybrid microfluidic device.

**Fig. 2:**
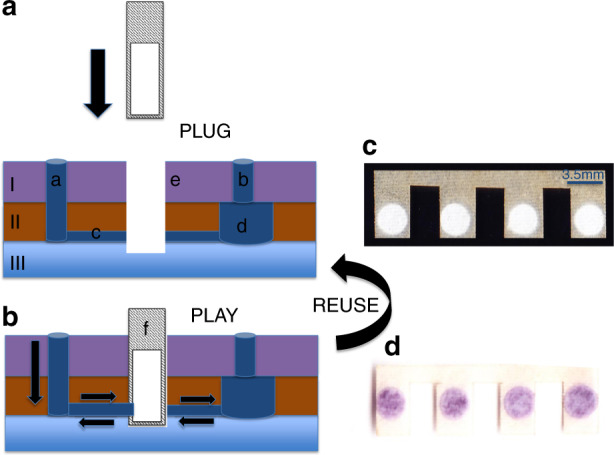
Working principle of the hybrid PnP microfluidic device. The figure shows the cross-sectional view of the hybrid PnP microfluidic device without the paper substrate (**a**) in the slot and with the paper substrate in the slot (**b**). The top layer consists of inlet microwells (a) and reservoir microwells (b). The middle layer consists of channels (c) and reservoirs (d). Slots (e) pass through the top, middle and half of the bottom layers where the paper substrate (f) can be inserted vertically. Scanned image of the SU-8-treated paper substrate before the immunoassay (**c**) and after the immunoassay (**d**).

Figure 2 illustrates the working principle of the PnP hybrid microfluidic device for ultrasensitive immunoassays using IgG, the most prevalent antibody in human circulation, as a model analyte. The top layer (I) has inlet microwells (a) from which reagents/analytes can be added. The top inlet microwell is connected to a microwell in the middle layer (II), which is located just below the inlet microwell so that reagents can flow directly into the middle layer. The microwell in the middle layer continues to the channels (c) in the middle layer through which reagents flow towards the reservoir (d). Along the channels (c), the reagent flows through the slots (e) where the paper substrate (f) is inserted vertically, as shown in Fig. 2b. The slot is fully open in the top and the middle layers, but it is only partially open on the top side of the bottom layer so that the paper strip can be inserted at a fixed location but the reagent and the paper substrate do not fall from the chip. The paper strip was pre-patterned with circular zones by using the SU8-based photolithography technique so that only reagents can pass through the circular hydrophilic region (white circles in Fig. 2c), which is connected with the PMMA microchannels, while other areas of the paper strip are hydrophobic. During the assay, a sample containing IgG is injected into the inlet microwells, passes through the paper substrate and is stored in the reservoir (d). The analyte IgG is then withdrawn back to the syringe pump connected to the inlet while passing through the paper strip. When passing through the paper strip, IgG is adsorbed onto the paper strip. The back-and-forth process is repeated several times so that a large amount of the analyte can be enriched on the paper. The enriched IgG will then go through the ELISA assay steps using 5-bromo-4-chloro-3-indolyl phosphate/nitro blue tetrazolium (BCIP/NBT) as the colour development substrate^25^, as illustrated in Fig. S1. When the sample repeatedly passes through the porous paper, the IgG in the entire sample can be efficiently adsorbed on the paper substrate/fibres, whereas conventional, static ELISA can only capture a small portion of the analyte because the majority of the analyte in the sample is far away from the immobilization substrate, although some of the analyte can migrate to the immobilization substrate (require a great deal of time). Thus, our hybrid PnP microfluidic device enables ultrasensitive colorimetric immunoassay without the aid of costly and high-sensitivity detectors, such as a fluorescence detector.

### Optimization of the flow rates

**Fig. 3:**
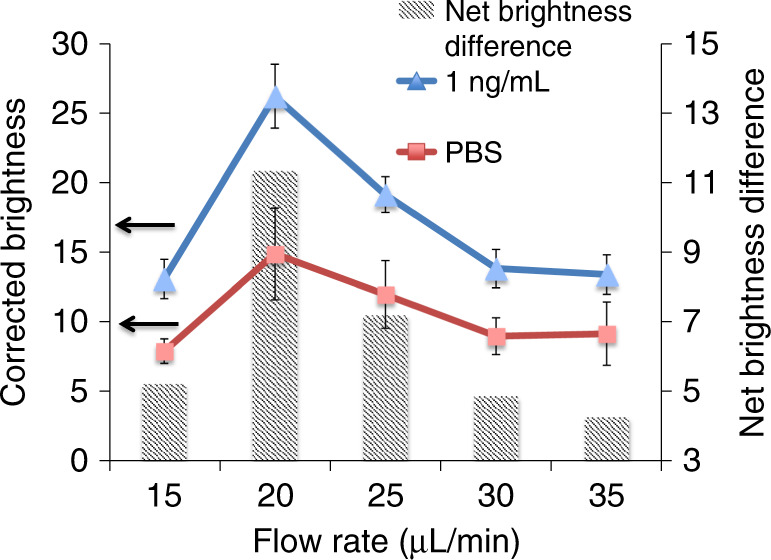
Optimization of the flow rate of the analyte. The line graph shows the corrected brightness values for ELISA of 1.0ng/mL IgG and the negative control (PBS) at different flow rates. The bar graph shows the net brightness difference between the analyte (IgG) and PBS at different flow rates.

The flow rate of a sample passing through the paper strip affects the immobilization or the enrichment efficiency of an analyte on the paper surface. Therefore, the optimum flow rate of the sample was determined using IgG as the model target in this optimization study. The ability to detect IgG levels in the human body is of great significance since it indicates the immune status of different diseases, including measles, mumps, rubella (MMR), hepatitis B virus, and varicella^34^. IgG can also be used for the diagnosis of neuromyelitis optica^35^ and autoimmune hepatitis^36^ and is the major constituent of the secondary immune response to different kinds of infectious agents^37^. During this optimization process, different flow rates of IgG (15, 20, 25, 30, and 35 µL/min) were applied to enrich the analyte, followed by ELISA. Since most colorimetric ELISA assays have difficulty detecting 1 ng/mL IgG, we used a low concentration, 1 ng/mL IgG, to optimize the conditions and to show the enhanced capacity of our new method. After the completion of ELISA, the paper substrate was scanned through a desktop scanner, and ImageJ was used to measure the brightness values of the detection zones. As shown in Fig. 3, there was a significant increase in the net brightness difference between the analyte (1 ng/mL of IgG) and the negative control (phosphate-buffered saline, PBS) when increasing the flow rate from 15 to 20 µL/min. The net brightness difference between the analyte and the negative control started to decrease when the flow rate was increased from 20 µL/min to 35 µL/min. At the lowest flow rate of 15 µL/min, the flow pressure may not have been strong enough for the entire analytes to properly pass through the paper substrate (i.e., only partial IgG passed through the paper strip). Therefore, IgG could not be efficiently enriched. However, at flow rates higher than 25 µL/min, we hypothesized that analyte molecules passed the paper substrate too fast and could not bind to the paper surface properly. Therefore, the flow rate of 20 µL/min, which gave the highest net difference between the analyte and the negative control, was considered as the optimized flow rate and was used in the following experiments.

### Optimization of the enrichment time and enrichment cycles

**Fig. 4:**
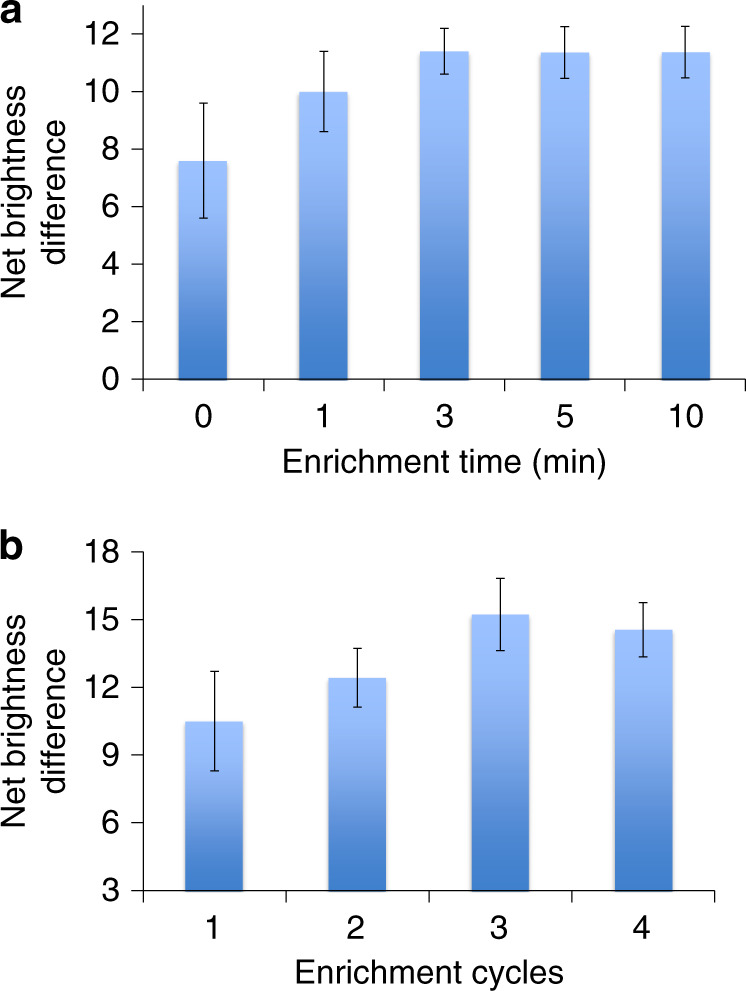
Optimization of enrichment time and enrichment cycle number for immunoassays utilizing the hybrid PnP microfluidic device. Enrichment time refers to the incubation time allowing for the analytes to be adsorbed on paper in a static status between each cycle of pumping through paper. **a** The relationship between the enrichment time and net brightness difference between the analyte and the negative control. **b** The relationship between the number of enrichment cycles and net brightness difference between the analyte and the negative control.

The enrichment time (incubation time allowing for analytes to be adsorbed on paper between each cycle of passing through the paper) and number of enrichment cycles affect the amount of analyte adsorbed onto the surface of the paper substrate. Hence, the optimum enrichment time and enrichment cycles were also determined. With the optimized flow rate of the analyte, different enrichment times (0, 1, 3, 5, and 10 min) were evaluated to optimize the enrichment time for the analyte IgG. As seen from Fig. 4a, there was a slight increase in the net brightness difference between the analyte and the negative control from 0 to 3 min. After 3 min of enrichment between each flow cycle, there was no significant increase in the net brightness difference. In addition, the standard deviation at 0 and 1 min was much higher than that at 3 min. The enrichment time of 3 min was considered optimal, as we could observe from Fig. 4a, where the net brightness difference from the ELISA colour against enrichment time showed a plateau after 3 min. Therefore, the enrichment time of 3 min was used in all subsequent experiments.

We also optimized the number of enrichment cycles for the analyte to determine the minimum enrichment cycles required to achieve maximum enrichment to increase the sensitivity in a minimum amount of time. The injection and withdrawal of the analyte were repeated for several enrichment cycles (1 cycle, 2 cycles, 3 cycles, and 4 cycles). As we observed from Fig. 4b, the net brightness difference between the analyte IgG and the negative control increased with the increasing number of enrichment cycles up to 3 cycles. After 3 cycles, there was no significant increase in the net signal, as the graph of the net brightness difference against enrichment cycles showed a maximum value at 3 enrichment cycles. Hence, the optimum number of enrichment cycles was considered 3 for all subsequent experiments.

### Ultrasensitive quantitative detection of IgG in the hybrid PnP microfluidic device

**Fig. 5:**
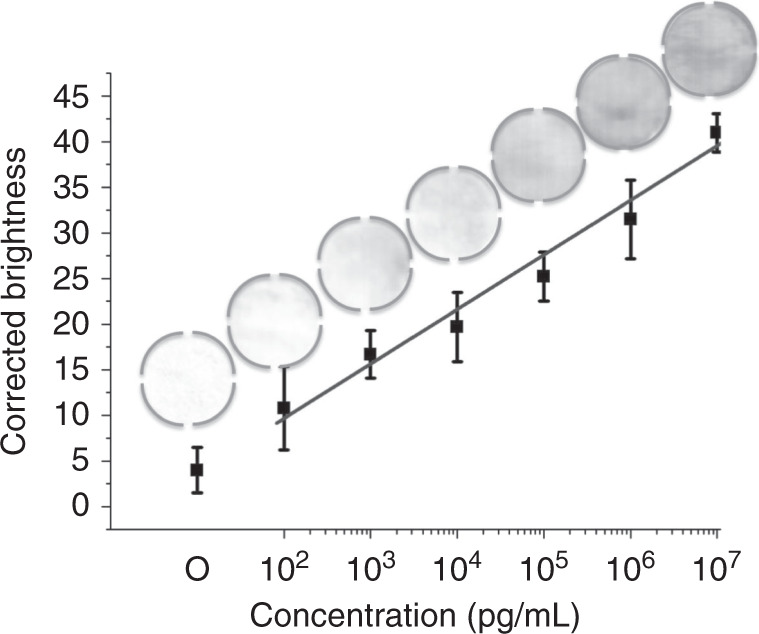
Sensitive detection of IgG in the hybrid PnP microfluidic device. Figure shows a linear plot of the corrected brightness of IgG over a concentration range from 10^2^ pg/mL to 10^7^ pg/mL. The inset shows the grey-scale image obtained by converting the RGB image of the paper substrate spotted with different IgG concentrations. RGB images were obtained by scanning the paper substrate with an office scanner.

To demonstrate the paper/polymer hybrid PnP microfluidic device for ultrasensitive immunoassays, we first performed the on-chip ELISA of IgG in our hybrid PnP microfluidic device under optimized conditions. Different concentrations of IgG ranging from 0.1 to 10 μg/mL were injected into the device using the syringe pump to perform the colorimetric ELISA. The result could be observed with the naked eye, and a portable desktop scanner was used to scan the paper strip. The average brightness of the signal from the scanned images was measured using ImageJ for quantitative analysis. Figure 5 shows the grey-scale image scanned by the desktop scanner for IgG immunodetection on the PnP hybrid microfluidic device along with its corresponding corrected brightness. The detection zone containing PBS had the brightest colour, and the detection zone containing 10^7^ pg/mL IgG had the darkest colour. The brightness decreased from 10^2^ pg/mL to 10^7^ pg/mL, from left to right in the figure. More importantly, the brightness of the hydrophilic detection zones was proportional to the concentration of IgG. A wide linearity range was found over the whole-concentration range from 10^2^ pg/mL to 10^7^ pg/mL with a linear regression of *y* = 5.30 log (*x*) + 15.72 (*R*^2^ = 0.99). It is worth noting that our PnP device exhibited an excellent, wide, dynamic range spanning 5 orders of magnitude (from 10^2^ to 10^7^ pg/mL). The LOD for IgG using the hybrid PnP microfluidic device was calculated to be 200 pg/mL based on a 3-fold standard deviation (SD) above the blank value. Therefore, our PnP device is ~10-fold more sensitive than commercial ELISAs utilized for the detection of IgG, which have LODs of 1.6–6.25 ng/mL^10^. Our hybrid PnP device (200 pg/mL) was also more sensitive than a paper-based device^21^, a modified PMMA device (0.12 μg/mL)^27^, a complementary metal oxide semiconductor device (10 ng/mL)^38^, and a paper/polymer hybrid microfluidic microplate (1.6 ng/mL)^23^. Table 1 lists the detailed comparison of ELISA detection of IgG using our PnP device with other devices.

**Table 1:** Comparisons among different devices for ELISA of IgG.

Device	Volume/well (Channel)	Total time	LOD	POC detection	Detection	Reagent addition	Reference
96-well plate ELISA	50–100 μL	18 h	1.6–6.25 ng/mL	☒	Spectrophotometer	Manual/Robotic pipettors	^10^
Paper-based device	3 μL	1 h	54 fmol/zone	☒	Scanner	Manual	^21^
Modified PMMA	30 μL	13 h	0.12 μg/mL	☒	(APTES modification) Fluorescence microscopy	Syringe pump	^27^
Complementary metal oxide semiconductor	100 μL	20+ h	10 ng/mL	☒	CCD camera	Manual	^38^
Paper/polymer Hybrid device	5 μL	1 h	1.6 ng/mL	☑	Scanner	Automatic	^23^
Our device	25 μL	70 min	0.2 ng/mL	☑	Scanner/smartphone	Syringe pump	

### Ultrasensitive ELISA detection of HBsAg in the hybrid PnP microfluidic device

Hepatitis B virus (HBV) is a major cause of chronic hepatic damage and hepatocellular carcinomas worldwide^39^, and ~30% of the world’s population shows serological evidence of current or past infection^40^. HBV affects more than 2 billion people worldwide, and there are still more than 350 million chronic carriers^41^. Twenty-five percent of people acquiring HBV infection in childhood develop primary liver cancer or cirrhosis when they grow up^42^. HBsAg, a serological biomarker for HBV infection, appears 2–10 weeks after exposure to HBV and persists beyond 6 months due to progression to chronic infection^40^. HBsAg can diagnose acute and chronic hepatitis B virus^43–45^, and the titer indicates the level of infection and severity of the disease^45,46^. The HBsAg level is highest in the immune-tolerant phase (4.5–5.0 log10 IU/mL), declines in the immune-clearance phase (3.0–4.5 log10 IU/mL) and decreases slowly and progressively after HBsAg seroconversion. The HBsAg level is lowest (1.5–3.0 log10 IU/mL) in individuals who maintain persistently normal serum alanine aminotransferase levels (low replicative phase), and it has been proposed that a reduction of HBsAg of ≥ 1.0 log10 IU/mL might reflect improved immune control. A concentration of 1 IU/mL is equivalent to 1–10 ng/mL HBsAg, which is, in turn, equivalent to 2 × 10^8^ subviral particles of HBsAg or 5 × 10^7^ virions^47^. Thus, HBsAg was used as another model biomarker to demonstrate the wide application of this hybrid PnP microfluidic platform.

**Fig. 6:**
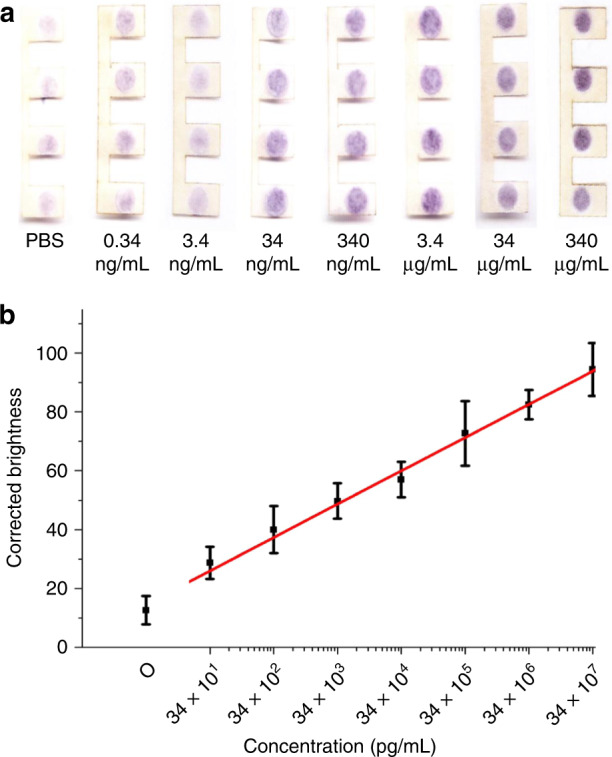
Ultrasensitive ELISA detection of HBsAg in the hybrid PnP microfluidic device. **a** Scanned images of the paper strips with different HBsAg concentrations by an office scanner. **b** Linear plot of the corrected brightness of HBsAg over a concentration range from 34 × 10^1^ pg/mL to 34 × 10^7^ pg/mL.

Figure 6a shows the colour images scanned by the desktop scanner for the ultrasensitive colorimetric ELISA detection of HBsAg from 0.34 ng/mL to 340 μg/mL in the PnP microfluidic device. The detection zone with PBS showed the brightest colour, and the zone with 340 μg/mL HBsAg showed the darkest purple colour. The signal intensity of the scanned image was calculated by ImageJ, and a calibration curve of HBsAg over a concentration range from 34 × 10^1^ to 34 × 10^7^ pg/mL was obtained (Fig. 6b). In addition, a wide linearity range was found over the whole-concentration range from 34 × 10^1^ to 34 × 10^7^ pg/mL with a linear regression of *y* = 10.89 log (*x*) + 33.16 (*R*^2^ = 0.99). The LOD of HBsAg using the hybrid PnP microfluidic device was found to be 270 pg/mL based on a 3-fold SD above the blank value. Our device was ~5-fold more sensitive than commercial ELISA kits utilized for the detection of HBsAg^48^.

### Comparison of the PnP device with other paper-based devices for the detection of HBsAg

**Fig. 7:**
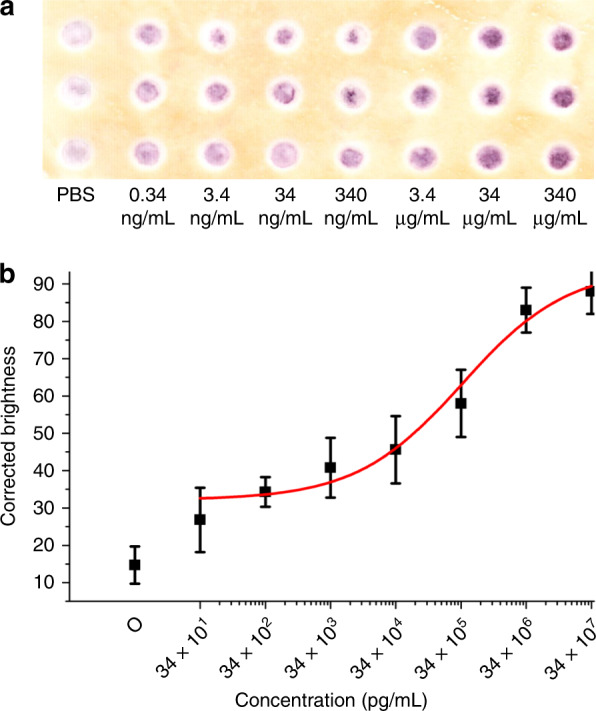
ELISA of HBsAg on a paper-based device. **a** Scanned image of the paper substrate with different HBsAg concentrations, which were acquired with an office scanner. **b** Linear plot of the corrected brightness of HBsAg over a concentration range from 34 × 10^1^ to 34 × 10^7^ pg/mL.

To further compare the sensitivity and the linear dynamic range of our PnP microfluidic method with other paper-based ELISA methods, ELISA of HBsAg was also performed in a regular paper-based device. Figure 7a shows the image scanned by the desktop scanner for HBsAg immunodetection on the paper-based device. Similar to the detection of HBsAg on the hybrid PnP microfluidic device, the intensity of the purple colour increased from 0.34 to 340 μg/mL, from left to right in the figure. However, the purple colour intensity only increased slightly from 0.34 ng/mL to 3.4 ng/mL and then became saturated at 34 μg/mL. The signal intensity from the scanned images was calculated using ImageJ as described before. Figure 7b is the calibration curve of HBsAg over a concentration range from 34 × 10^1^ pg/mL to 34 × 10^7^ pg/mL. Unlike the hybrid PnP device, the linearity range was only found to be between 34 × 10^2^ pg/mL and 34 × 10^5^ pg/mL with a linear regression equation of *y* = 7.80 log (*x*) + 28.73 (*R*^2^ = 0.96). The LOD of HBsAg using the paper-based device was found to be 2.9 ng/mL, which was 10-fold higher than that of the hybrid PnP device. In addition, the repeated washing steps required in ELISA led to a decrease in hydrophobicity of the hydrophobic barrier on the paper surrounding the hydrophilic zone, as indicated by slightly white regions around the hydrophilic zone after the assay (Fig. 7a). The damaged hydrophobic barrier further resulted in the spreading of the reagents from the hydrophilic zones over the hydrophobic area, leading to cross-contamination. Finally, as seen from Fig. 7a, analytes and colour production in the paper-based device tended to agglomerate at the centre of the hydrophilic zone. In contrast, the adsorption of the analyte and, hence, the colour production in the PnP hybrid microfluidic device were distributed more evenly (Fig. 6a) compared to those in the paper-based devices. In the absence of external force, water tends to form droplets because of the surface tension and become agglomerated at the centre of the hydrophilic zone; thus, the analytes were adsorbed mostly at the centre and concentrated the area of colour production. The tendency to form droplets increased with increasing storage time of the SU-8 fabricated paper substrate because the hydrophilic zones became more hydrophobic. In contrast, the repeated back-and-forth flow of the sample through the paper strip in the PnP device facilitated even distribution of the analyte and colour production, thus increasing the uniformity of protein absorption.

Surprisingly, we observed that the paper-based device offered a dynamic range of 3 orders of magnitude (34 × 10^2^–34 × 10^5^ pg/mL), whereas the PnP hybrid microfluidic device exhibited a much wider dynamic range of 6 orders of magnitude (34 × 10^1^–34 × 10^7^ pg/mL), which was an increase of 3 orders of magnitude in the linear dynamic range. To gain insight into the reason, we also investigated the enzyme kinetics to qualitatively understand the wide linear dynamic range in the PnP hybrid device compared to that of the paper-based device. Alkaline phosphatase acts on the BCIP/NBT substrate to convert it to NBT diformazan following Michaelis-Menten kinetics. The concentration of enzyme [E] used in ELISA is much less than the substrate concentration [S]. Therefore, the rate of product [P] formation is given by

1 $$\upsilon = \frac{{d\left[ P \right]}}{{{\mathrm{dt}}}} = V_{{\mathrm{max}}}\frac{{[S]}}{{K_M + [S]}} = k_{{\mathrm{cat}}}[E]_0\frac{{[S]}}{{K_M + [S]}}$$

where *V*_max_ = maximum rate, *K*_M_ = the Michaelis constant, *k*_cat_ = catalytic rate, and [*E*]_0_ = initial enzyme concentration^49^.

The order of reaction depends upon the size of the two terms in the denominator (*K*_M_ and [*S*]) when the substrate concentration is much higher than the enzyme concentration. In the paper-based device, all of the substrate, [*S*]_paper_, is added at once, but in the PnP hybrid microfluidic device, the substrate, [*S*]_PnP_, flows slowly through the syringe. Therefore, at any given time, the effective concentration of the substrate in the PnP hybrid device [*S*]_PnP_ < [*S*]_paper_, i.e., the substrate concentration in the paper-based device. From Eq. (1), at a low substrate concentration in the PnP hybrid device, [S] ≪ *K*_M_ and $\upsilon = k_{{\mathrm{cat}}}[E]_0\frac{{[S]}}{{K_M}}$. Thus, the reaction rate varies linearly with the substrate concentration [S], which is first-order kinetics and thus leads to a larger linear range. However, in paper-based devices, all of the substrate is added at once, leading to a high substrate concentration [S], so [*S*] ≫ *K*_M_, and the reaction is independent of [S], implying zero-order kinetics, which reaches its maximum rate *V*_max_ = *K*_cat_ [*E*]_0_. The reaction becomes saturated, and further addition of substrate does not increase the rate of reaction, leading to a small range of linearity.

## Conclusions

We developed a simple, eco-friendly, and portable hybrid PnP microfluidic device for ultrasensitive immunoassay, where the results of the assay could be viewed within 70 min with the naked eye or scanned by a simple desktop scanner for quantitative analysis. Without the use of any specialized equipment, the LODs of 200 pg/mL for IgG and 270 pg/mL for HBsAg were achieved, which were at least 10-fold better than those using commercial ELISA kits^10^. In addition, the hybrid device showed a wide linear range of five orders of magnitude for IgG and six orders of magnitude for HBsAg.

This hybrid device takes advantage of both paper and PMMA substrates, providing a unique and low-cost platform for carrying out colorimetric assays of different biomolecules with high detection sensitivity. The hybrid PnP microfluidic device has four important features compared to regular paper-based or PMMA microfluidic devices: (i) the entire sample passes back-and forth through the paper substrate with a certain flow rate enriching the amount of analytes, ultimately increasing the sensitivity of the device so that low-concentration analytes, which is typical in real-world samples, can be measured; (ii) the paper substrate can be replaced with a new substrate after completion of the assay so that the main PMMA framework can be reused multiple times in this PnP microfluidic device; (iii) reagents/analytes can be easily and rapidly transferred through the microchannels to the paper substrate, avoiding the slow flow utilized in paper-based devices; and (iv) due to the unique introduction of the 3D micro-porous paper substrate with a high surface-to-volume ratio to the PMMA device, proteins can be rapidly immobilized within a few minutes compared to overnight incubation in traditional microplates, which also avoids the complicated surface modification of PMMA. Additionally, images can also be captured by smartphone cameras and analysed using different applications or cloud-based systems for on-site quantitative detection^50^. Therefore, the hybrid PnP microfluidic device can be used for ultrasensitive and quantitative detection of infectious diseases, cancers, and other biomolecules, particularly for resource-poor settings, including rural areas, small clinics, border regions, and developing countries, where expensive diagnostic equipment, such as microplate readers, is not easily available.

## Experimental section

### Microfluidic platform design and fabrication

The PnP hybrid microfluidic platform was designed with the aid of Adobe Illustrator CS5. The PMMA chip was then fabricated with a laser cutter (Epilog Zing 16, Golden, CO) by mask-less laser ablation, where the beam of the laser moved in the x- and y-directions according to the chip design. The final design of the PnP hybrid device is shown in Fig. 1. The hybrid PnP microfluidic device had varying depths in each of the PMMA layers. Fabricating such a device through photolithography methods requires multi-level microfabrication and alignment along with multiple photomasks, which makes the process much more time-consuming, complicated and expensive^51,52^. Thus, we used laser ablation as a rapid alternative to photolithography, where the laser removes the material from the desired substrate according to the pattern^53^.

A reversible sealing of the PnP device was obtained by clamping different PMMA layers together within glass slabs in an oven at 115–120 °C for 35 min. The PnP device was used for enrichment and assays after plugging the paper substrate into the slots after the chip cooled to room temperature.The PMMA device can be reused for multiple immunoassays after washing with 70% ethanol followed by excess water and PBS buffer with 1% bovine serum albumin.

### Optimization of parameters affecting colorimetric signals in the hybrid device

ELISAs of different biomarkers were performed in the hybrid PnP microfluidic device. Various factors, including the flow rate, enrichment time, and enrichment cycles, influence the sensitivity of ELISA. A thorough study was performed to optimize the conditions of ELISA to obtain better sensitivity. Finally, the ELISA detection of IgG and HBsAg was performed under optimized conditions.

#### Optimization of the flow rates in the hybrid device

To determine the relationship between the flow rate and signal intensity, ELISA of IgG was performed with different flow rates. Samples/reagents were injected into the microwells by polyethylene tubing (BD Diagnostics, Sparks, MD) connected to a syringe pump (kdScientific, Holliston, MA) to control the flow rate. IgG (1 ng/mL) and the negative control (PBS) were added to each inlet microwell in the first layer. To optimize the flow rate of the analyte, ELISA was performed with different flow rates (15, 20, 25, 30, and 35 µL/min). Fifty microliters of the analyte/PBS was added to the chip with these different flow rates (so that each micro-zone received 25 µL of the analyte). Once 50 µL of the analyte was added and incubated for 5 min, the analyte was withdrawn back to the syringe so that it flowed through the paper surface within the channel. This process was repeated 5 times to maximize enrichment of the analyte onto the paper surface. After the completion of the colorimetric assay, the flow rate with the highest net brightness (signal) difference between the analyte and the negative control was taken as the optimum flow rate with which further experiments were performed.

#### Optimization of the enrichment time and enrichment cycles in the hybrid device

Enrichment time was defined as the time of incubation after the analyte was completely injected or withdrawn from the PnP hybrid microfluidic device in each enrichment cycle. The enrichment time was optimized similar to the optimization of the flow rate. Once the analyte was completely injected into the device or withdrawn from the device using the optimum flow rate, the paper substrate was incubated for different time periods (0, 1, 3, 5, and 10 min), and the process was repeated for five cycles. Finally, the colorimetric assay was completed to measure the net brightness difference between the analyte and the negative control. For the optimization of the enrichment cycles, the complete injection and withdrawal of the analytes were repeated for several cycles (1, 2, 3, and 4 cycles) with the optimized flow rate and enrichment time. The signal intensities were compared against enrichment cycles after the assay, and the enrichment cycle with the highest net brightness difference between the analyte and the negative control was considered the optimum enrichment cycle number.

### **Detection of IgG and HBsAg using the hybrid PnP microfluidic device**

The hybrid PnP device was used for the detection of IgG and HBsAg. Figure S1 shows a schematic illustration of the colorimetric ELISA of IgG in the PnP hybrid device. Standard samples of IgG were prepared by diluting a stock solution of IgG (2 mg/mL in 10 mM, pH 8.0 PBS) to different concentrations ranging from 0.1 ng/mL to 10 μg/mL. Fifty microliters of IgG/PBS was injected into the hybrid PnP device from different inlet microwells in the top layer of the chip. The analyte was injected from the syringe pump via polyethylene tubing to the inlet microwells at a speed of 20 µL/min. The device was incubated for 3 min, and then the analyte was withdrawn back to the syringe pump at the same speed of 20 µL/min followed by an enrichment time of 3 min. The injection and withdrawal of the analytes were repeated for three cycles with the same speed and enrichment time. After the enrichment step, the paper surface was blocked with a blocking buffer (4.5% bovine serum albumin (BSA) and 0.05% Tween-20 prepared in PBS at pH 7.4) to block the non-specific binding sites. After incubating for 10 min, the device was washed with washing buffer (PBST; 0.05% Tween 20 in 10 mM, pH 7.4 PBS). Then, 6 μg/mL of anti-rabbit IgG linked with alkaline phosphatase was added for 7 min, which was followed by three washes with PBST. A light yellow-coloured BCIP/NBT (5-bromo-4-chloro-3-indolyl phosphate/nitroblue tetrazolium), which is a substrate for alkaline phosphatase, was then added. The substrate produces a visually observable purple colour, the intensity of which depends on the concentration of the analyte. Finally, the paper substrate was pulled out of the device after 10 min, and the paper substrate’s side that was facing the inlet wells could be scanned with a scanner or an image could be taken by a cell-phone camera.

For the on-chip enrichment and detection of HBsAg, a sandwich immunoassay was performed with a procedure similar to that of IgG detection. HBsAg was first bound to the surface of the paper strip, followed by blocking of the unreacted surfaces. Next, anti-HBsAg was added, followed by the addition of anti-rabbit IgG, which was linked with alkaline phosphatase. Unbound secondary antibody in the sandwich structure was then washed with PBST, and, finally, the colorimetric substrate BCIP/NBT was added.

### **Comparison of the PnP device with paper-based devices for the detection of HBsAg**

For the detection of HBsAg in a paper-based device, a 40-microzone paper substrate with an array (8 × 5) of circular zones was prepared as discussed in section 1.2 of the SI, and colorimetric ELISA detection of HBsAg at concentrations ranging from 0.34 ng/mL to 340 μg/mL was performed. First, 10 μL of different concentrations of HBsAg was added to each microzone and incubated for 10 min, followed by blotting the paper-based device dry with a kimwipe (Kimtech, Roswell, GA). The paper substrate was then blocked with a blocking buffer (5 μL per zone) for 10 min and washed with PBST. The paper-based device was blotted dry after each step. Anti-HBsAg was then added for 10 min followed by washing and the addition of ALP-linked anti-rabbit IgG. Finally, BCIP/NBT was added after washing three times with PBST. The paper-based device was scanned with a desktop scanner for quantitative measurement.

## Supplementary information

SI
