# Supplementary material for: A reusable PMMA/paper hybrid plug-and-play microfluidic device for an ultrasensitive immunoassay with a wide dynamic range
Source: Microsyst Nanoeng. 2020 Jun 15;6:28. doi: 10.1038/s41378-020-0143-5 (PMC8433292; doi:10.1038/s41378-020-0143-5)
Supplement: Supplementary file 1 — SI [file 41378_2020_143_MOESM1_ESM.docx]

Supporting Information:

A Reusable PMMA/Paper Hybrid Plug-and-Play Microfluidic Device for Ultrasensitive Immunoassay with Wide Dynamic Range

Sharma T. Sanjay,^1 †^ Meihan Li,^1 †^ Wan Zhou,^1^ Xiaochun Li,^2^ and XiuJun Li ^1,3^*

^1:^ Department of Chemistry and Biochemistry, University of Texas at El Paso, 500 West University Ave, El Paso, Texas, 79968, USA

^2:^ College of Biomedical Engineering, Taiyuan University of Technology, Taiyuan, Shanxi 030024, China

^3:^ Border Biomedical Research Center, Biomedical Engineering, and Environmental Science and Engineering, University of Texas at El Paso, 500 West University Ave, El Paso, Texas, 79968, USA

Corresponding Author: Dr. XiuJun Li. E-mail: [xli4@utep.edu](mailto:xli4@utep.edu)

^†^ denotes equal contribution to the work.

**1. MATERIALS AND METHODS**

**1.1. Chemicals and Materials:**

IgG from rabbit serum, Tween 20, albumin from bovine serum, anti-rabbit IgG-alkaline phosphatase, BCIP/NBT liquid substrate, and Phosphate Buffer Saline were purchased from Sigma Aldrich (St. Louis, MO). HBsAg protein (subtype ad) was purchased from Fitzgerald Industries International Inc., Acton, MA. Polyclonal anti-HBsAg was purchased from Novus Biologicals, Littleton, CO. PMMA was purchased from McMaster-Carr, Los Angeles, CA. Whatman #1 chromatography paper was purchased from Sigma Aldrich (St. Louis, MO). Ultrapure Milli-Q water (18.2 MΩ.cm) obtained from a Millipore water purification system (Bedford, MA) was used in all assays and reagent preparation, unless otherwise noted.

**1.2. Fabrication of paper-based devices:**

The paper-based device was fabricated using a photolithography method.^1^ Photoresist (SU-8) was first added to chromatography paper and evenly spread on both sides of the paper. The paper was then baked in the hot plate pre-heated to 135 ^0^C for 20 min (10 min on each side). Then, the paper substrate was placed below a pre-designed plastic photomask and exposed to UV light for 30 sec. UV light polymerized the photoresist. The paper was incubated again on the hot plate at 135 ^0^C for 5 min (2.5 min on each side). Finally, the paper substrate was washed with acetone followed by isopropyl alcohol to wash off un-polymerized photoresist to give hydrophilic zones. Polymerized regions could not be washed off and form the hydrophobic regions. The paper based-device was used after drying.

**1.3. Data analysis**. After the completion of the assay, the paper substrate was scanned with a simple desktop scanner (CanoScan LiDE 700F, Canon). ImageJ software that is distributed for free by NIH (<http://rsb.info.nih.gov/ij/download.html>) was used to measure the average brightness of the hydrophilic test zone in the paper substrate. In the RGB (red + green + blue) image obtained, the signal of the individual hydrophilic zone was calculated as the average of the brightness intensity of respective pixels. The software converts the RGB image into gray scale using the formula gray = (red + green + blue) ⁄ 3. ImageJ has gray values ranging from 0 to 255, from minimum (darkest) to maximum (brightest). Corrected brightness was used in the study to represent the graph and for data analysis. To obtain the corrected brightness, an average brightness value from data was subtracted from the maximum value i.e., 255.


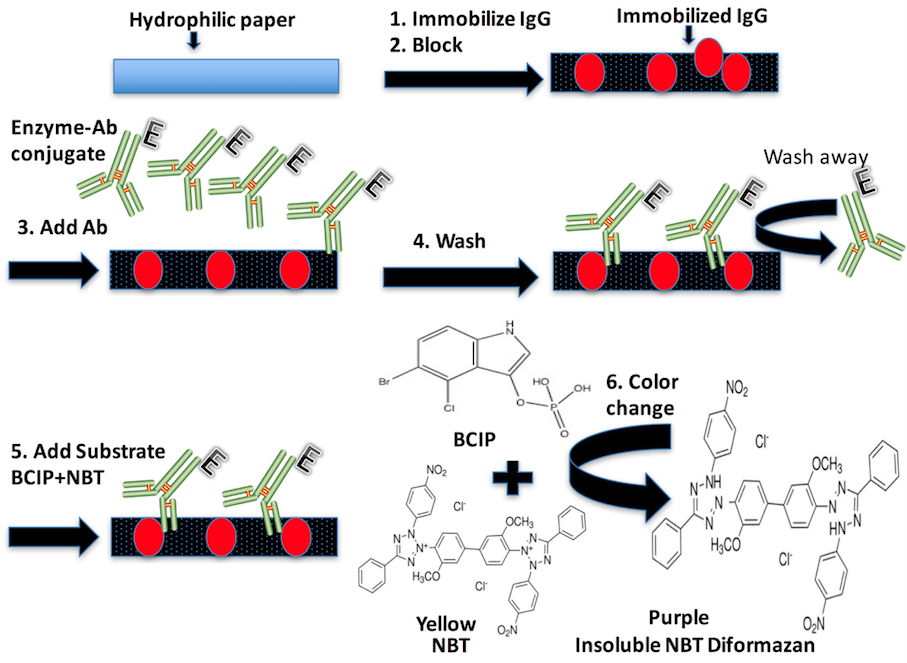


Figure S1. Schematic illustration of colorimetric ELISA of IgG in PnP hybrid microfluidic device: (1) Immobilization of IgG on the patterned paper substrate, (2) Blocking by using 4.5 % BSA, (3) Washing with PBST and addition of the enzyme-conjugated antibody, (4) Washing with PBST, and (5) Addition of the substrate for the production of purple insoluble NBT diformazan.

**Reference:**

[1] Martinez, A. W., Phillips, S. T., Wiley, B. J., Gupta, M., & Whitesides, G. M., *Lab on a Chip*, **2008**, 8, 2146.
